# Siliceous spicules in a vauxiid sponge (Demospongia) from the Kaili Biota(Cambrian Stage 5), Guizhou, South China

**DOI:** 10.1038/srep42945

**Published:** 2017-02-21

**Authors:** X.-L. Yang, Y.-L. Zhao, L. E. Babcock, J. Peng

**Affiliations:** 1 College of Resource and Environment Engineering, Guizhou University, Guiyang 550025, China; 2 State Key Laboratory of Palaeobiology and Stratigraphy, Nanjing Institute of Geology and Palaeontology, CAS, Nanjing 210008, China; 3 School of Earth Sciences, The Ohio State University, Columbus 43210, USA

## Abstract

Fossils of the sponge *Angulosuspongia sinensis* from calcareous mudstones of the middle and upper part of the Kaili Formation (Cambrian Stage 5) in the Jianhe area of Guizhou province, South China, exhibit an apparently reticulate pattern, characteristic of the Vauxiidae. Energy Dispersive X-Ray Spectrometry (EDS) and Raman spectroscopy analysis indicate the presence of silica in the skeletal elements of these fossils, suggesting that this taxon possessed a skeleton comprised of spicules. This is the first confirmation of siliceous skeletal elements in fossils of the family Vauxiidae, and it lends support to the hypothesis that some early demosponges possessed biomineralized siliceous skeletons, which were subsequently lost and replaced by spongin later in the evolutionary history of this lineage. The new materials provide critical insight into the phylogeny and evolution of biomineralization in the Demosopongiae.

The earliest stages of animal evolution remain highly unclear^1^. Recent studies of molecular genetics suggest that sponges lay near the base of the animal phylogenetic tree^2^,^3^,^4^. Whether the sponges are monophyletic or polyphyletic is subject to interpretation^2^. Based on aspects of cellular structure and chemistry, some workers have favored the possibility that sponges have multiple origins from two or three different single-celled ancestors^5^,^6^. Sponges certainly have a deep evolutionary history^7^. Articulated body fossils and isolated spicules have been reported from the Ediacaran^8^,^9^,^10^,^11^,^12^ and the earliest Cambrian^13^,^14^,^15^,^16^. Biomarker evidence suggests that sponges evolved even earlier, perhaps as early as the Cryogenian Period^17^. The majority of Proterozoic fossils interpreted as sponges, however, have been questioned^18^, owing to the presumed non-spicular nature of some^19^, which would have resulted in a poor fossil record of the group^20^. Thus, while fossils of early aspiculate sponges are unusual, they are essential in unraveling sponge origins and in understanding demosponge phylogeny and the history of spicule evolution.

To date, the best-known early aspiculate poriferans are those assigned to the family Vauxiidae Walcott, 1920, which is known primarily from the Cambrian. The Vauxidae are characterized an apparently reticulate, aspiculate fibrous skeleton^21^. The skeletal composition of the Vauxidae has been reinterpreted a number of times^21^,^22^,^23^,^24^,^25^,^26^, but recently, Ehrlich *et al*.^27^ confirmed chitin in the skeleton of *Vauxia gracilenta*, and concluded that it was a “keratose” demosponge rather than mineralized spicules, and proposed that the Vauxiidae were likely to be the most basal definitive demosponge group known. This contrasts with a recent hypothesis that some aspiculate sponge skeletons were derived evolutionarily through demineralization of siliceous spicules and loss of spicules may have happened at least twice in the Demospongiae^20^. Botting *et al*.^28^ identified spicules within skeletal strands of *Vauxia bellula* from the Cambrian Burgess Shale. On the basis of this evidence, Botting *et al*.^29^ suggested that diactines were primitive for some Keratosa, and that spicules predate the appearance of the demosponge crown group and were subsequently lost in Myxosongiae and Keratosa.

This study seeks to test the loss of spicules hypothesis in order to shed light on the major pathways in the skeletal evolution of Demosopongiae and the skeletal composition of the Vauxiidae. For this reason we have studied a new genus and species, *Angulosuspongia sinensis*^30^, a vauxiid sponge from the middle and upper part of the Kaili Formation (Cambrian Series 3, Stage 5) of Jianhe, Guizhou, South China (Fig. 1). The skeletal elements of the taxon consistently show a hexagonal to slightly irregular, quadrangular–hexagonal architecture (Figs 1e,f, 2a,b and 3a,b), which similar to forms from the Burgess Shale of British Columbia, Canada^21^, and the Spence Shale and Wheeler formations of Utah, USA^25^. The Jianhe material illustrates apparent spicular structures, composed of silica and preserved in relief, which were previously unknown in the Vauxiidae. Although spicules were previously described in *Vauxia bellula*^28^ the original mineralogy of its spicules is unkown. The Jianhe specimens add an interesting dimension to the evolutionary history of the Demospongea, as they clearly indicate that some early Paleozoic ancestors of nonbiomineralizing ‘Keratosa’ possessed siliceous spicules. The new material lends support to the hypothesis that some early demosponges possessed biomineralized siliceous skeletons, and later in their evolutionary history, silica was replaced by spongin.

## Results

### Skeletal composition

**Figure 1:**
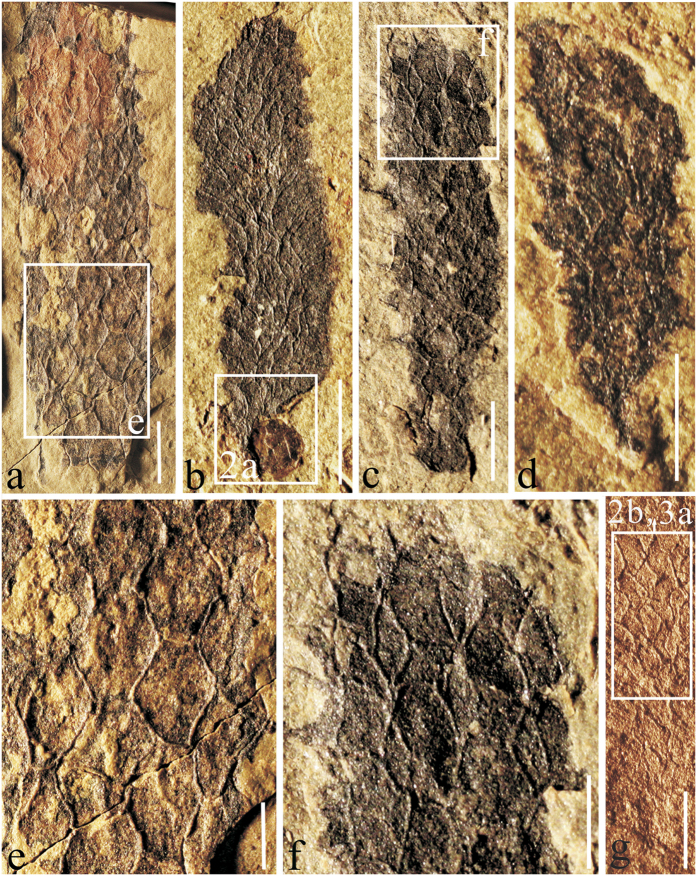
*Angulosuspongia sinensis*, a vauxiid sponge from the Kaili Biota. (**a**) GTBM16-109a; (**b**) GTBM9-4-4598a, rectangular areas are enlarged in Figs 2a and 3d,e, showing a brachiopod preserved together with the root of a vauxiid sponge; (**c**) GTBM9-2-4792a; (**d**) GTBM17-1761b, specimen analyzed by Raman spectroscopy; (**e**) close-up view of the rectangular area in Fig. 1a, showing the hexagonal openings produced by fused spicules; (**f**) close-up view of the rectangular area in Fig. 1c, showing the polygonal openings produced by fused spicules; (**g**) GTBM16-1192, specimen gold-coated for SEM analysis, rectangular area is enlarged in Figs 2b and 3a–c; Scale bars equal 2 mm in (**a**–**c**), 1mm in (**d**–**g**). All specimens photographed dry in reflected light. The original source of the photo in Fig. 1g comes from Geological Magazine^30^.

Taphonomic characteristics of the vauxiid sponge *Angulosuspongia sinensis* from the Kaili Biota suggest a rather rigid skeletal network, in contrast to flexible fibers comprised of spongin or chitin. Skeletons of Jianhe materials, although compacted in mudstone, are consistently preserved three-dimensionally, with robust rays extending beyond the margin of the sponge body (Figs 1 and 3a). This manner of preservation indicates that the skeletal elements were capable of resisting flattening during sediment compaction.

Energy-dispersive X-ray spectroscopy (EDS) and elemental mapping were used to determine the composition of skeletal elements. The analyses reveal O and Si to be major components in both the sponge body and rock matrix, but the sponge spicules have far less Al, K and Fe than rock matrix, and the sponge body is enriched in C relative to the matrix (Fig. 4; Table 1). It is worth noting that skeletons were more silicon-rich and with less carbon than the inner of polygonal openings produced by fused spicules (Fig. 4a,b,d,e). Raman spectroscopy analysis (Fig. 5) indicates that the vauxiid sponge skeletons of the Kaili Biota are composed of kerogen and silica and demonstrates that the kerogen is composed of geochemically moderately altered amorphous carbonaceous matter (interlinked polycyclic aromatic hydrocarbons) like that of other Burgess Shale-type fossils of Cambrian age^31^,^32^.

Confirmation of the presence of SiO_2_ in the spicular skeleton provides strong support for the interpretation that this species possessed siliceous spicules. It is unlikely that silica is a secondary diagenetic product in specimens of vauxiid sponges of the Kaili Biota, as co-occurring, biomineralized brachiopods do not show evidence of mineral replacement (Fig. 4j–o). As is typical of the Burgess Shale-type preservation, high-fidelity preservation of labile soft tissues in fossils of the Kaili biota resulted primarily from conservation of primary organic remains, as two-dimensional carbonaceous (kerogen) films^32^. These films are sometimes augmented by early diagnenetic mineralization by pyrite or apatite rather than silicification or aluminosilicification^33^,^34^.

## Discussion

Prior to the description of the Jianhe materials, the family Vauxiidae was monogeneric, embracing only *Vauxia*. In *Vauxia* and putatively related sponges, three interpretations of skeletal composition have emerged.

1. Siliceous spicule hypothesis. Walcott^22^, and later de Laubenfels^23^ classified *Vauxia* as a hexactinellid sponge. Finks^24^ regarded *Vauxia* as a specialized offshoot of the hexactinellid Protospongiidae. Implicit in this classification is a skeletal composition of opaline silica. Walcott^22^ reported that in all specimens he examined from the Burgess Shale of British Columbia, Canada, the original siliceous matter of the spicules was removed and replaced by pyrite or a black carbonaceous material, or a combination of the two.
2. Keratose spongin fiber hypothesis. Rigby^25^ reassigned the family Vauxiidae to the lithistid Demospongea on the basis of skeletal symmetry. He stated that the symmetry of *Vauxia* is more similar to the symmetry of the Demospongea than the Hexactinellida. Later, Rigby^26^ concluded that the vauxiid skeleton was probably comprised of spongin fibers rather than biomineralized spicules, and Rigby and Collins^21^ characterized the skeleton as having a double-layer construction of apparently fused keratose fibers. According to Ehrlich *et al*.^35^ spongin in poriferans results from a hierarchical, multilevel organization of collagen microfibrils. The collagen microfibrils are densely packed, and arranged in a preferential orientation, usually in concentric layers.
3. Spongin-chitin hypothesis. Ehrlich *et al*.^35^ demonstrated the presence of α-chitin in spongin fibers of two extant demosponges of the order Verongida (*Aplysina* sp. and *Verongula gigantea*). Chitin, or poly [β(1→4)-2-acetamido-2-deoxy-D-glucopyranose], is a polymer of the aminosugar *N*-acetylglucosamine, and is often associated with proteinaceous skeletons in invertebrates. Ehrlich *et al*.^35^ also identified calcium carbonate in the form of aragonite in the two verongiids, and stated that it is responsible for the stability of the sponge skeleton. Later, Ehrlich *et al*.^27^ identified the presence of chitin in *Vauxia gracilenta* from the Burgess Shale.

Maldonado^20^ hypothesized that spongin skeletons evolved at least twice in the Demospongiae. Spicules, which he considered to have been siliceous, were, in his view, lost and replaced by spongin fibers at least once in the Chondrosida-Verongida lineage, and also in the Haplosclerida - Dictyoceratida - Dendroceratida lineage. The skeletal composition of the Jianhe specimens are intriguingly consistent with Walcott’s^22^ interpretation that *Vauxia* from the Burgess Shale originally possessed siliceous spicules that were later replaced, and offer support for Maldonado’s^20^ hypothesis that spongin fibers replaced siliceous spicules in some demosponge lineages. These findings support the view that spicules were present among skeletal strands of *Vauxia bellula*^28^ and confirm their originally siliceous composition. This new information does not entirely refute Rigby’s^26^ interpretation of the Vauxiidae as non-biomineralized, as siliceous spicules appear to have been present in some species, but not others. The simultaneous occurrence of spicules and spongin fibers in vauxiid sponges may also support Botting’s hypothesis of a single origin of spicules prior to the appearance of crown-group Silicea, and the subsequent loss of spicules in early ‘keratosan’ sponges^28^,^29^ rather than the traditional and molecular-based views of demosponge phylogeny^36^.

## Methods

**Figure 2:**
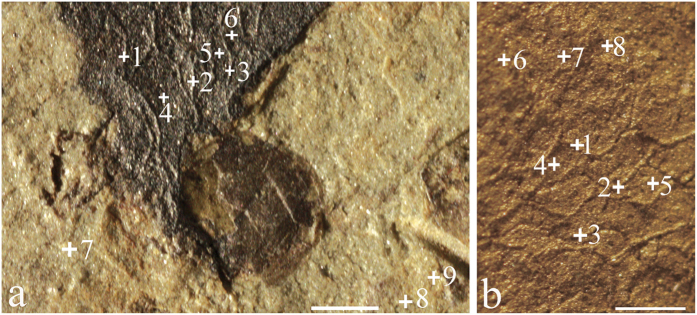
(**a**) Close-up view of the rectangular area in Fig. 1b; (**b**) close-up view of the rectangular area in Fig. 1g. Crosses denote EDS points, crosses 1-3 in Fig. 2a,b mark sponge spicules, crosses 4-6 in Fig. 2a and 4–5 in Fig. 2b mark the inner of polygonal openings produced by fused spicules, and crosses 7–9 in Fig. 2a and 6–8 in Fig. 2b mark the surrounding matrix. All scale bars equal 0.5 mm. All specimens were photographed dry in reflected light.

**Figure 3:**
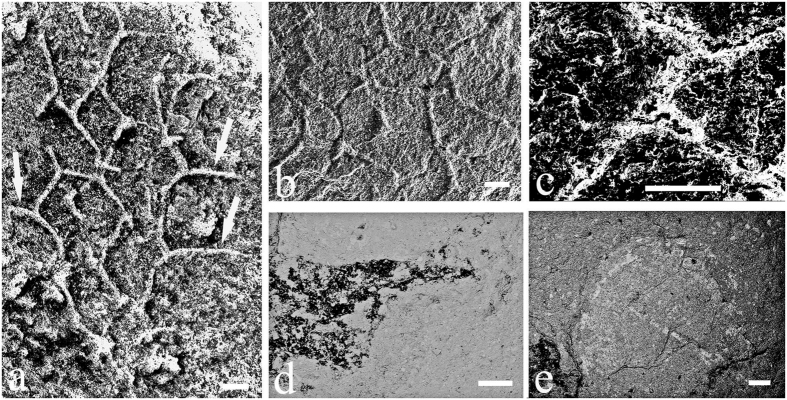
SEM images under high-vacuum of skeletons of *Angulosuspongia sinensis* from the Kaili Biota. (**a**–**c**) Secondary electron (SE) images of close-up view of the rectangular area in Fig. 1g (GTBM16-1192), and arrows showing robust rays extending beyond the margin of the sponge body; (**d**–**e**) Backscattered electron (BSE) images of close-up view of the rectangular area in Fig. 1b (GTBM9-4598a), (**d**) root of sponge, (**e**), brachiopod preserved together with vauxiid sponge. All scale bars equal 200 μm. Thanks to Yan Fang from Nanjing Institute of Geology and Palaeontology for taking these photos. The original source of the photo in Fig. 3a comes from Geological Magazine^30^.

**Figure 4:**
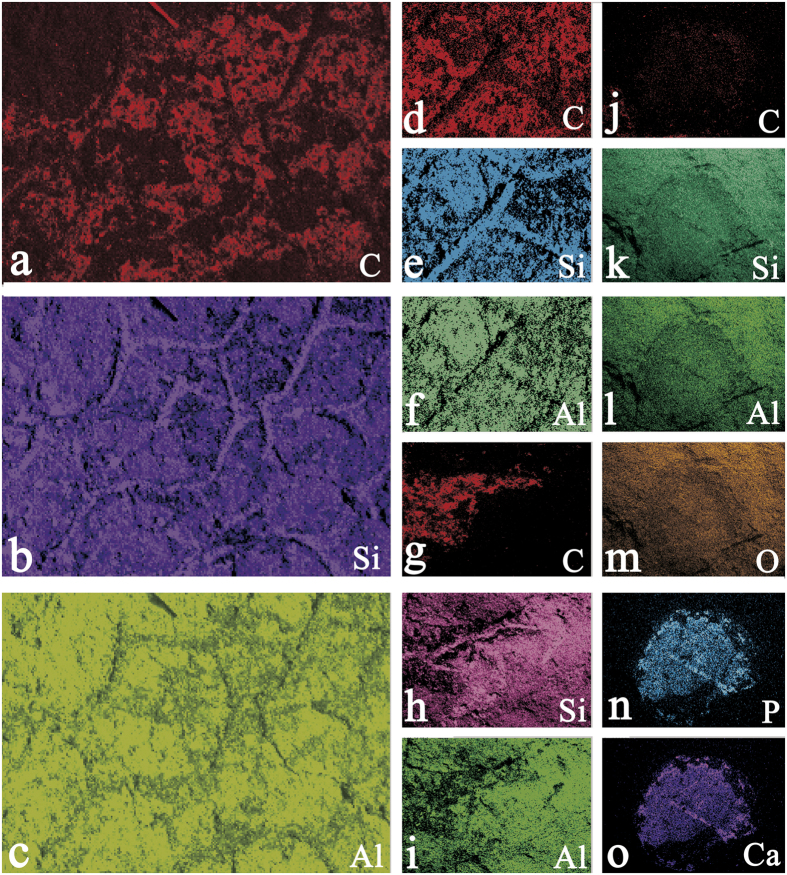
Elemental mapping analyses. (**a**–**c**) elemental map of Fig. 3b; (**d**–**f**) elemental map of Fig. 3c; (**g**–**i**) elemental map of Fig. 3d; (**j**–**o**) elemental map of Fig. 3e. Thanks to Yan Fang from Nanjing Institute of Geology and Palaeontology for taking these photos.

**Figure 5:**
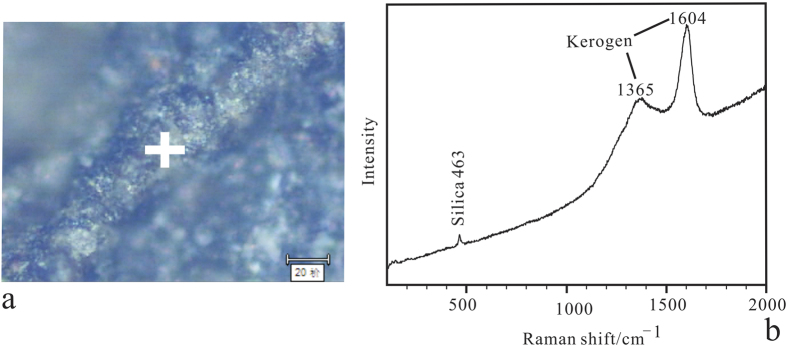
Overlapping Raman spectra showing the major bands of silica and kerogen that comprise the spicule of *Angulosuspongia sinensis*. (**a**) Raman spectroscopy image of spicule of GTBM17-1761b; (**b**) Raman spectrum taken from the cross in Fig. 5a. Thanks to Yuning Yang from Northwest University for taking these photos.

Sixty-two specimens of vauxiid sponge remains were examined in this study. They are housed at the Guizhou Research Center for Paleontology, Guizhou University, Guiyang, China (GRCP, GU). Some specimens (Figs 1 and 2) were imaged using a Canon EOS Rebel T3i Digital SLR camera with MP-E 65 mm macro lens. Others (Figs 3 and 4) were imaged at finer scale using a LEO1530VP Scanning Electron Microscope (SEM) equipped with an Energy-dispersive X-ray Spectrometer (EDS), located in the State Key Laboratory of Palaeobiology and Stratigraphy, Nanjing Institute of Geology and Palaeontology, Chinese Academy of Sciences. One sample, GM-16-1192 was gold-coated, whereas GTBM9-4598a imaged under the SEM were left uncoated. Raman spectroscopy analyses for skeletal composition of vauxiid sponges from the Kaili Biota were performed on an Invia model Raman spectrograph of the Renishaw company. In this instrument, two laser devices with wavelengths of 514 nm and 785 nm excite monochromatic light. Experiment conditions: optical laser wavelengh was 514 nm, scanned area was 100 to 2000 cm^−1^, time of exposure was 10 s.

## Additional Information

**How to cite this article**: Yang, X.-L. *et al*. Siliceous spicules in a vauxiid sponge (Demospongia) from the Kaili Biota(Cambrian Stage 5), Guizhou, South China. *Sci. Rep.* **7**, 42945; doi: 10.1038/srep42945 (2017).

**Publisher's note:** Springer Nature remains neutral with regard to jurisdictional claims in published maps and institutional affiliations.

## Figures and Tables

**Table 1:** Energy dispersive X-ray spectroscopy (EDS) point analyses of *Angulosuspongia sinensis*.

	*C*	*O*	*Mg*	*Al*	*Si*	*K*	*Ca*	*Fe*	*S*
Fig. 2a-1	47.65	27.88	0.18	1.57	19.97	0.60	0.59	0.70	0.87
Fig. 2a-2	44.98	30.84	0.38	0.61	22.83	0.12	0.04	0.20	
Fig. 2a-3	10.49	40.96	0.46	1.08	46.32	0.36	0.12	0.21	
Fig. 2-4	38.74	28.49	1.52	3.75	23.75	1.34	0.22	2.18	
Fig. 2-5	47.30	26.96	1.71	4.27	10.71	1.53	0.26	6.67	0.58
Fig. 2a-6	71.70	6.59	1.18	4.15	8.31	1.83	0.32	5.35	0.56
Fig. 2a-7	5.71	45.40	2.20	10.99	20.04	2.64	0.43	12.78	
Fig. 2a-8	10.68	39.98	1.97	11.10	20.55	8.12	0.40	7.20	
Fig. 2a-9	13.97	41.13	2.71	10.75	21.06	3.26	1.21	5.92	
Fig. 2b-1	21.95	26.64	0.71	1.36	47.12	0.45	0.36	1.39	
Fig. 2b-2	26.86	28.70	0.70	1.99	39.31	0.50	0.47	1.48	
Fig. 2b-3	34.25	30.29	0.85	1.83	30.60	0.54	0.60	1.04	
Fig. 2b-4	38.08	23.16	0.78	3.01	30.36	0.99	1.05	2.56	
Fig. 2b-5	56.25	19.17	1.00	4.25	10.95	1.44	1.28	5.66	
Fig. 2b-6	12.71	30.76	2.74	14.22	27.00	6.39	0.31	5.87	
Fig. 2b-7	12.08	30.79	1.50	13.71	28.04	7.41	0.55	5.92	
Fig. 2b-8	17.84	23.98	2.21	11.32	29.85	4.81	0.51	9.47	

EDS points are marked on Fig. 2. Elemental compositions are shown in estimated weight percentages.
